# Pediatric antiphospholipid syndrome: clinical features and therapeutic interventions in a single center retrospective case series

**DOI:** 10.1186/s12969-022-00677-8

**Published:** 2022-02-23

**Authors:** Jacqueline A. Madison, Kelsey Gockman, Claire Hoy, Ajay Tambralli, Yu Zuo, Jason S. Knight

**Affiliations:** 1 Department of Pediatrics, Division of Pediatric Rheumatology, University of Michigan, Ann Arbor, MI USA; 2 Department of Internal Medicine, Division of Rheumatology, University of Michigan, 1150 West Medical Center Drive, Ann Arbor, MI 48109 USA

**Keywords:** Pediatrics, Antiphospholipid syndrome, Antiphospholipid antibodies, Thrombosis, Systemic lupus erythematosus, Damage Index (DIAPS), Non-criteria manifestations, Anti-phosphatidylserine/prothrombin

## Abstract

**Background/purpose:**

Pediatric antiphospholipid syndrome (APS) is a thromboinflammatory disease characterized by the presence of circulating antiphospholipid antibodies and either thrombotic events or pregnancy morbidity. The objective of this study was to review a large institution’s experience to better understand the characteristics of children with APS.

**Methods:**

We conducted a retrospective review of pediatric APS at a tertiary referral center. The electronic medical record system was queried from 2000 through 2019, and 21 cases were included based on meeting the revised Sapporo Classification criteria by age 18 or younger. Comparisons between primary and secondary APS patients were made with two-tailed t-tests.

**Results:**

Twenty-one patients were included with a median age at diagnosis of 16 years and median follow-up of 5.8 years. Secondary APS was slightly more common than primary APS (11 vs. 10 cases) and was primarily diagnosed in the context of systemic lupus erythematosus. Two thirds of patients (67%) also had “non-criteria” manifestations of APS including thrombocytopenia, autoimmune hemolytic anemia, and livedo reticularis/racemosa. Almost half of patients (43%) had recurrent thrombosis, typically when patients were subtherapeutic or non-adherent with anticoagulation. Damage Index in Patients with Thrombotic APS (DIAPS) scores indicated a chronic burden of disease in both primary and secondary APS patients.

**Conclusion:**

This case series of pediatric APS provides important context regarding disease phenotypes displayed by children with APS. High prevalence of non-criteria clinical manifestations highlights the need to consider these characteristics when developing pediatric-specific classification criteria and when considering this relatively rare diagnosis in pediatric practice.

## Background

Antiphospholipid syndrome (APS) is a systemic autoimmune disease characterized by thrombotic events and/or pregnancy morbidity in the setting of persistently positive antiphospholipid antibodies (aPL). In adults, the Sapporo criteria (first developed in 1999 and revised in 2006) are used to formally classify APS for research purposes. These criteria require the presence of at least one clinical event (venous, arterial, or small vessel thrombosis, or pregnancy-related morbidity) and the durable presence over at least 12 weeks of at least one laboratory feature: positive lupus anticoagulant (a functional assay that screens for aPL), anticardiolipin IgG or IgM in medium or high titer (> 40 GPL/MPL or titer > 99^th^ percentile), or anti-beta-2 glycoprotein I (β_2_GPI) IgG or IgM (titer > 99^th^ percentile) [1]. There are no pediatric-specific criteria. Studies have shown that up to 25% of otherwise healthy children may have low levels of aPL, which may be transient and related to the developing immune system’s response to antigens, whether infectious or nutritional [2]. The concept of pediatric APS has generally been applied to children age 18 and younger, although other cut-offs including age 16 and 21 have also been used [3]. There is an ongoing effort to develop new APS classification criteria, including criteria specific for pediatric patients with APS [4].

The largest pediatric cohort described to date consists of an international registry of 121 patients diagnosed with APS before age 18, while the largest case series of North American pediatric APS patients included 17 children diagnosed at age 18 and younger [5, 6]. There have been a few other retrospective case series that are small in number but add to the characterization of this disease [7–11]. A consistent theme of these series is the presence of non-criteria manifestations: non-thrombotic clinical features not included in the updated Sapporo criteria such as livedo reticularis or racemosa, persistent thrombocytopenia, autoimmune hemolytic anemia, choreiform movements, white matter changes, cardiac valve abnormalities, and more [2, 6, 12, 13]. A task force on APS clinical features developed evidence-based recommendations for future criteria to include APS nephropathy, heart valve lesions, thrombocytopenia, livedo reticularis, chorea, and longitudinal myelitis [14]. There are few data in pediatric patients regarding non-criteria lab tests, such as anti-phosphatidylserine/prothrombin (anti-PS/PT) antibodies, a class of antibodies regularly associated with lupus anticoagulant positivity and thrombotic events [15–17].

Beyond challenges in diagnosis and characterization of pediatric APS, little is known about organ damage accumulated over time. Recently, the disease-specific Damage Index in Patients with Thrombotic APS (DIAPS) has been proposed to assess for damage accrual, although it has yet to be applied to a pediatric population [18, 19]. The objective of this study was to gather additional information about clinical characteristics, laboratory aPL positivity over time, treatments employed, thrombotic outcomes, and damage accrual in pursuit of enabling a more personalized and proactive approach to the care of children with APS.

## Methods

### Cohort identification and analysis

The University of Michigan IRB approved this study (HUM00161442). The electronic medical record system of Michigan Medicine was queried for patients aged 21 and younger with a diagnosis of APS from 2000 through 2019. Fifty cases were evaluated further, and 21 were ultimately included in this study. Excluded cases failed to meet the revised Sapporo Classification criteria by age 18 or younger. Of the 29 excluded cases (19 female and 10 male; 20 White or Caucasian, 7 Black or African-American, 1 Hispanic, and 1 Asian-American), 8 (28%) were excluded due to age over 18 at the time of meeting criteria (typically age of thrombotic event), and the remainder were excluded due to failing to meet the classification criteria at any point: 5 (17%) due to lack of laboratory criteria, 5 (17%) due to lack of clinical criteria, and 11 (38%) with neither laboratory nor clinical criteria (for example, a patient with a family history of APS in the electronic medical record system). Included cases were assessed for clinical and laboratory features, therapeutic management, and outcome data. Disease manifestations were identified by searching notes and imaging reports. One included case did not strictly meet Sapporo Classification criteria as the patient was treated aggressively with plasmapheresis and rituximab with resolution of traditional aPL tests by the time they were repeated six months later; this patient did, however, have durably positive non-criteria aPL (anti-PS/PT). There were no cases of neonatal APS or definite catastrophic APS (CAPS) identified in this cohort.

### Antiphospholipid antibody measurements

Prior to 2015, aPL levels (anti-β_2_GPI and anticardiolipin) were measured at Michigan Medicine by Werfen QUANTA Lite® ELISA (maximum = 150). Since 2015, aPL have been quantified via a multiplex assay using the BioPlex 2200 System (maximum = 112). Neither the Werfen nor BioPlex assays utilize biotin-streptavidin reagents and thus should not be confounded by patient biotin levels or exogenous biotin use. A few laboratory test results were obtained from outside laboratories for which we do not know the details regarding the specific tests used. The lupus anticoagulant panel done at Michigan Medicine includes prothrombin time (reference range 9.4 – 12.2 s) with INR, partial thromboplastin time (reference range 21.0–29.0 s), dilute Russell’s viper venom test (DRVVT, reference range < 44.0 s), DRVVT ratio (reference range < 1.3), and hexagonal phospholipid neutralization (reference range less than or equal to 6.0 s). The phosphatidylserine/prothrombin antibody IgG and IgM panel is an ELISA (Werfen) with a reference range of negative at less than or equal to 30.0 units, borderline between 30.1 and 40.0 units, and positive at greater than or equal to 40.1 units.

### Other autoantibody measurements

When available, we captured positivity of other autoantibodies. At Michigan Medicine, ANA is done by immunofluorescence assay (IFA) with a positive result at a titer of 1:80 or greater. Anti-double-stranded-DNA is done via chemiluminescent immunoassay; the current reference range is < 27 IU/mL = negative, 27–35 IU/mL = borderline positive, and greater than or equal to 36 IU/mL = positive; these cut-off values have changed slightly over time, and positives are reported as abnormal according to reference values at the time of testing. Anti-Sm and anti-chromatin testing is via an extractable nuclear antibody panel for which the test methodology is a multiplex flow immunoassay.

### Damage Index in Patients with Thrombotic APS (DIAPS)

This scoring system has recently been proposed to assess the accumulation of damage related to APS. The DIAPS adds APS-specific items to others taken from the SLICC/ACR Damage Index (SDI) for a total of 37 items: 22 from the SDI plus 15 new items [19]. The score is a simple summation of each output item. Severe damage has been suggested as a DIAPS score of 3 or more [20]. DIAPS has previously been used in patients with both primary APS and APS secondary to SLE, and different patterns in the kinetics of damage accumulation were identified in each group [21].

### Statistical analysis

Comparisons between primary and secondary APS groups were analyzed via two-tailed t tests using GraphPad software. Significance was defined as *p* < 0.05.

## Results

### Patient characteristics and clinical manifestations

Over a 20-year period, a total of 21 cases were identified. All met APS classification criteria by age 18 or younger. Within this cohort, there were 10 and 11 patients with primary and secondary APS, respectively. Among patients with secondary APS and a concomitant rheumatic disease, most (9 or 82%) had a diagnosis of systemic lupus erythematosus (SLE), and there was 1 patient each with ulcerative colitis and microscopic polyangiitis. The demographics and disease features of all of the pediatric APS patients identified are detailed in Table 1. The median age at diagnosis was 16 years (range 8–18 years). Overall, there were 16 females (76%) and 5 males (24%) with a similar distribution within the primary and secondary APS groups.

**Table 1:** Demographics and clinical manifestations of pediatric APS patients

	All APS (*n* = 21)		Primary APS (*n* = 10)		Secondary APS (*n* = 11)		*p* value^a^
**Median Age at Diagnosis**	16	(8–18)	16	(12–18)	16	(8–18)	0.42
**Sex**							
Female	16	(76%)	8	(80%)	8	(73%)	0.70
Male	5	(24%)	2	(20%)	3	(27%)	0.70
**Race/Ethnicity**							
White or Caucasian	17	(81%)	7	(70%)	10	(91%)	0.23
Black or African-American	3	(14%)	2	(20%)	1	(9%)	0.49
Hispanic	1	(5%)	1	(10%)	0	(0%)	0.29
**Clinical Manifestations**							
Obstetric	1	(5%)	0	(0%)	1	(9%)	0.34
Thrombotic	20	(95%)	10	(100%)	10	(91%)	0.34
Venous	13	(62%)	8	(80%)	5	(45%)	0.11
Arterial	6	(29%)	1	(10%)	5	(45%)	0.079
Small vessel	5	(24%)	3	(30%)	2	(18%)	0.54
Catastrophic APS	0	(0%)	0	(0%)	0	(0%)	
**Non-criteria Manifestations**							
Thrombocytopenia	11	(52%)	6	(60%)	6	(55%)	0.52
AIHA	9	(43%)	4	(40%)	6	(55%)	0.80
Livedo	5	(24%)	3	(30%)	2	(18%)	0.54
White matter lesions	3	(14%)	1	(10%)	2	(18%)	0.60
Seizure	3	(14%)	1	(10%)	2	(18%)	0.60
APS nephropathy	2	(10%)	1	(10%)	1	(9%)	0.95
Skin ulcer	1	(5%)	0	(0%)	1	(9%)	0.34
Valve abnormality	1	(5%)	1	(10%)	0	(0%)	0.29
Cognitive changes	1	(5%)	0	(0%)	1	(9%)	0.34
MS-like features	1	(5%)	0	(0%)	1	(9%)	0.34
**Laboratory Manifestations**							
Anti-β2-glycoprotein I	14	(64%)	8	(80%)	7	(64%)	0.42
Anti-cardiolipin	17	(81%)	9	(90%)	7	(64%)	0.16
Lupus anticoagulant	11	(52%)	6	(60%)	6	(55%)	0.80
Triple positive	10	(48%)	6	(60%)	4	(36%)	0.29
ANA	9	(43%)	0	(0%)	9	(82%)	**0.00020**
Anti-double-stranded DNA	10	(48%)	1	(10%)	9	(82%)	**0.0013**
Anti-chromatin	8	(38%)	1	(10%)	7	(64%)	**0.014**
Anti-Sm	3	(14%)	0	(0%)	3	(27%)	0.082
**Recurrent Events**	9	(43%)	4	(40%)	5	(45%)	0.81

^a^Comparing primary and secondary APS by unpaired t-test or Chi-squared test

*AIHA* Autoimmune hemolytic anemia, *MS* Multiple Sclerosis

Most patients in the cohort (95%) had a thrombotic event though one 18-year-old secondary APS patient was diagnosed with obstetric manifestations. Among those with thrombotic APS, most had venous thrombotic events (62%), while about a quarter had an arterial (29%) or small-vessel event (24%). More arterial events were seen in patients with secondary APS (5 compared to 1). Arterial events in this series included stroke, myocardial infarction, and other infarctions including those involving spleen and kidney. None of the patients in this cohort were diagnosed with CAPS. Regarding non-criteria manifestations, there were no significant differences between the primary and secondary APS groups. In our cohort, over half of patients (52%) had persistent thrombocytopenia (< 100,000/µl) at some point in their disease course. Autoimmune hemolytic anemia was also common, noted in 43% of patients. Livedo reticularis or racemosa was seen in 24% of patients. Other manifestations are detailed in Table 1.

### Laboratory manifestations

**Fig. 1:**
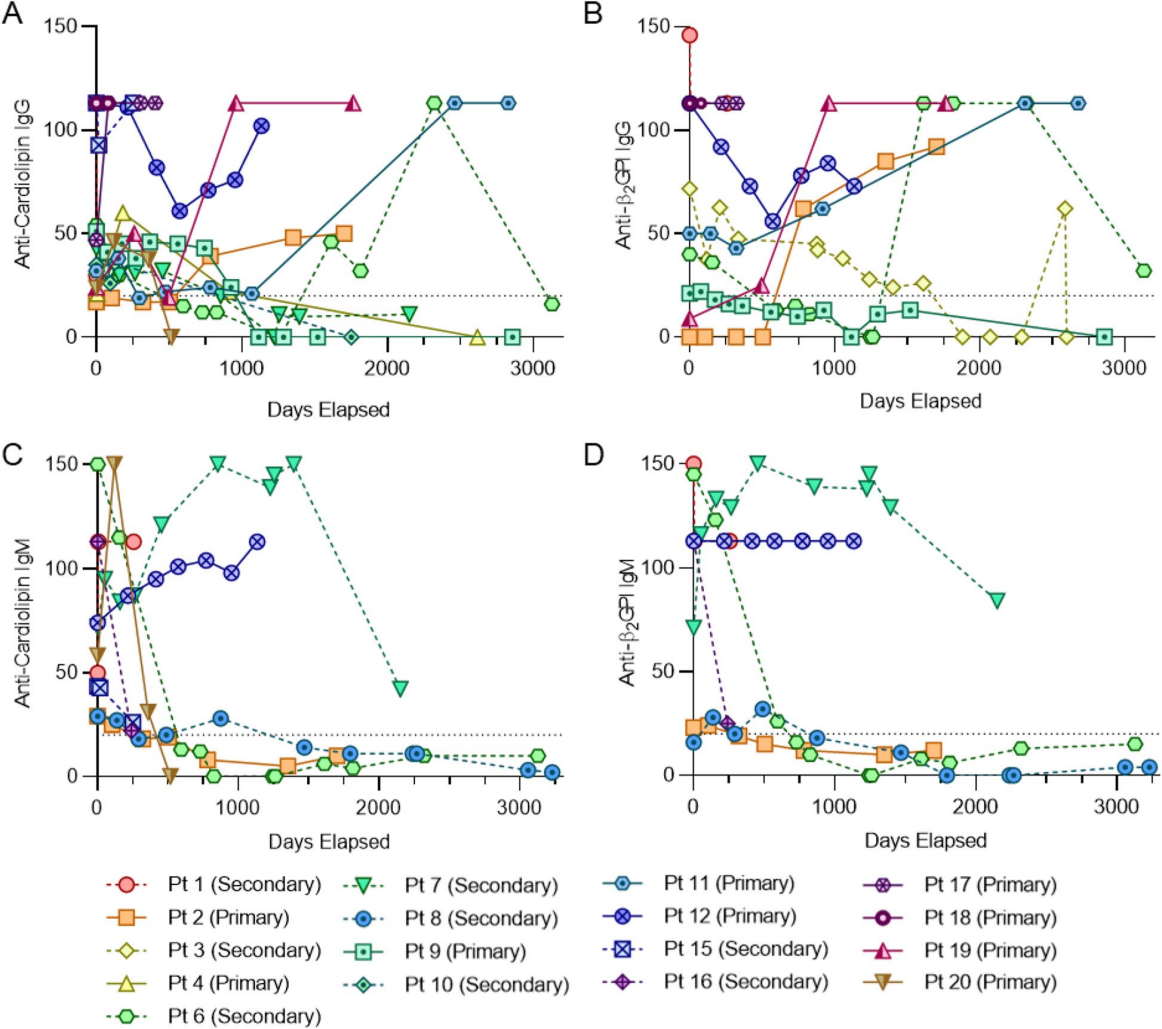
Trend in antiphospholipid antibody levels over time. **A,** Anticardiolipin IgG over time. **B,** Levels of anti-β2-glycoprotein I IgG over time. **C,** Anticardiolipin IgM over time. **D,** Anti-β2-glycoprotein I IgM over time. In all panels, the dotted horizontal line represents the level above which the result was identified as positive. To be included in this figure, a patient needed durability of an antiphospholipid antibody across at least 12 weeks apart

In considering the laboratory manifestations of APS, we evaluated the persistent positivity of typical aPL. Over at least 12 weeks, 64% of our cohort were positive for anti-β_2_GPI antibodies, 81% for anticardiolipin antibodies, and 52% for lupus anticoagulant. It should be noted that some patients did not have repeat lupus anticoagulant testing performed. Notes indicate that some providers may have avoided testing due to concomitant use of heparin products which may lead to challenges in the interpretation of the lupus anticoagulant assay depending on the assay, heparin formulation, and anti-factor Xa effect achieved. There were no statistically significant differences between the primary and secondary APS groups with regards to positivity of aPL. About half of the patients in the cohort (10 total, 48%) were triple positive for all three aPL tests. We also evaluated positivity of anticardiolipin and anti-β_2_GPI over time (Fig. 1). A variety of patterns were noted: continued positivity, increases in titer, and decreases in titer (some below the threshold of positivity), which could be either transient or persistent. There did not appear to be notable differences in patterns displayed among patients with primary versus secondary APS or among those with different types of thrombosis (venous, arterial, or small vessel). It did appear that IgG aPL were more likely to be durably positive as compared with IgM aPL, though we cannot draw definite conclusions given our small sample size. There was also no clear relationship between which patients were on hydroxychloroquine and those whose antibody titers decreased over time (data not shown).

In two patients (one each in the primary and secondary APS groups), there was positive testing for anti-PS/PT antibodies; we did not observe any negative tests in the cohort. In the patient with primary APS, the anti-PS/PT remained durably positive, even when anticardiolipin and anti-β_2_GPI antibodies became negative. Lupus anticoagulant was not tested regularly in this patient due to long-term use of low-molecular-weight heparin. We also tracked autoantibodies seen frequently in lupus; antinuclear antibodies (ANA), anti-double-stranded-DNA antibodies, and anti-chromatin antibodies were significantly more common in the secondary APS group and only rarely positive in the primary APS group (Table 1). Anti-Sm was detected only in the secondary APS group.

### Treatments

**Fig. 2:**
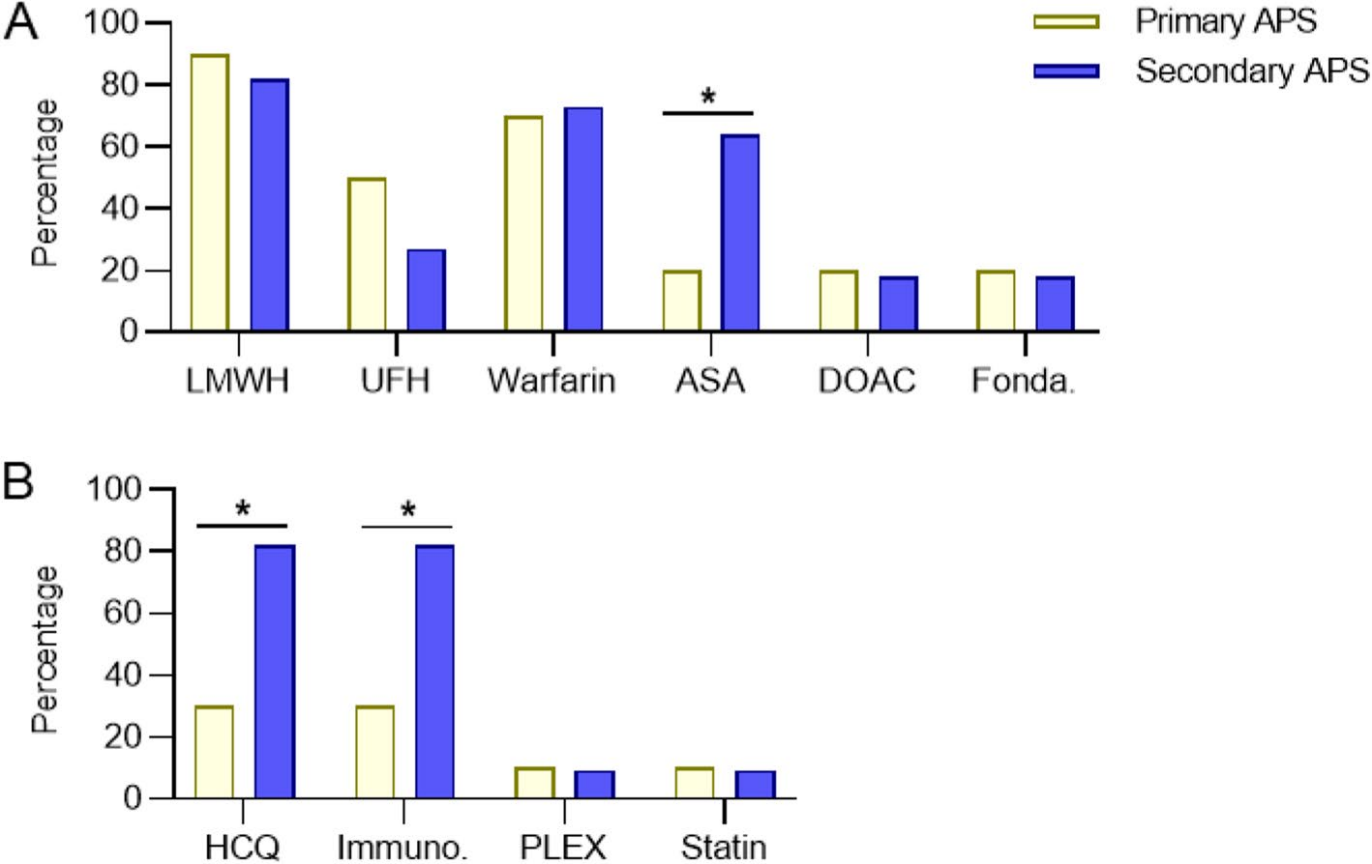
Treatments used in primary and secondary pediatric APS patients. **A,** Various anticoagulant and antiplatelet medications were employed. **B,** Other notable treatments. LMWH = low molecular weight heparin; UFH = unfractionated heparin; DOAC = direct oral anticoagulant; Fonda. = fondaparinux; HCQ = hydroxychloroquine; Immuno. = immunomodulatory therapy; PLEX = plasmapheresis

All patients were treated with some form of antiplatelet or anticoagulant therapy. Only one patient was treated with aspirin alone for the duration of follow-up of this study. As seen in Fig. 2, significantly more patients with secondary APS as compared with primary APS were treated with aspirin (*p* = 0.04). A small number of patients in both groups were treated with a direct oral anticoagulant or with fondaparinux. Not surprisingly, significantly more secondary APS patients were treated with hydroxychloroquine (*p* = 0.02) and with an immunomodulatory agent (*p* = 0.02). Primary APS patients who received immunomodulatory therapy (3 total) received some combination of rituximab (3), glucocorticoids (2), eculizumab (1), and IVIG (1). In the secondary APS group, a wider variety of immunomodulatory agents were prescribed for 9 patients: glucocorticoids (6), belimumab (3), mycophenolate mofetil (3), azathioprine (3), cyclophosphamide (2), methotrexate (2), quinacrine (2), rituximab (2), IVIG (1), tocilizumab (1), and abatacept (1). A small number of patients in both primary and secondary APS groups were treated with plasmapheresis or a statin.

### Outcomes

**Fig. 3:**
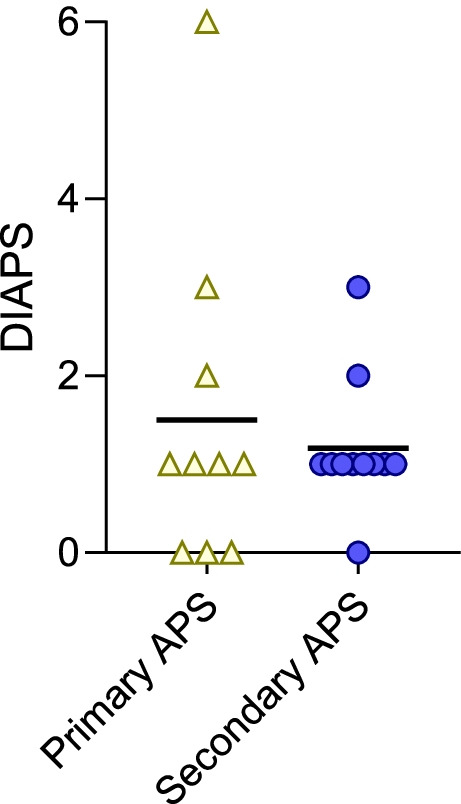
Damage index in Patients with Thrombotic APS (DIAPS) score in primary and secondary APS. The score was calculated at the time of each patient’s most recent follow-up appointment

Recurrence of thrombotic events was relatively common in both primary (40%) and secondary (45%) APS groups (Table 1). Although some had the same type of thrombotic event with their recurrence (such as a second episode of venous thrombosis), this was not always the case (Table 2). Almost all the patients with a recurrent event were either not prescribed therapeutic anticoagulation (aspirin alone) or were subtherapeutic on their anticoagulation, in some cases attributed to chance and in others noncompliance. One recurrent event occurred while a patient was on a direct oral anticoagulant (DOAC) albeit with questionable adherence, and a second occurred about a week after a patient stopped taking a DOAC due to hospitalization. Another recurrent episode of thrombosis occurred while a patient was receiving the combination of aspirin and fondaparinux. To quantify damage accrued over time, the DIAPS was utilized based on the clinical status at the patient’s most recent contact with their physician (Fig. 3). In both primary and secondary APS groups, damage measured via the DIAPS demonstrated similar median scores in the two groups (1.5 and 1.2, respectively). A total of 3 patients had a score of 3 or higher.

**Table 2:** Features and treatment of patients with recurrent thrombotic or obstetric events

**Pt**	Age first event (years)	APS type	Triple + aPL	First event	Initial treatment	Time to recurrence (months)	Recurrent event	AC prescribed at time of recurrence	Adherence to AC treatment
**5**	14	2°, Thr	Yes	Splenic infarction^a^	LDA	7.6	Renal TMA^b^	LDA	Yes
**8**	17	2°, Thr	Yes	Myocardial infarction^b^	Fondaparinux and LDA	80.5	Cardiac thrombus^d^	LDA; apixaban recently discontinued	Yes
**11**	16	1°, Thr	Yes	PE, lower extremity DVT^c^	Warfarin	59.0	Lower extremity DVT^c^	Warfarin	No (loss of health insurance)
**13**	16	2°, Thr	No	Lower extremity DVT^c^	Warfarin (planned for 3 months)	19.4	Lower extremity DVT^d^	None	-
						62.1	Lower extremity DVT^d^	Rivaroxaban	No (patient reported)
**14**	18	2°, Obs	No	HELLP, Pre-eclampsia, thrombotic vasculopathy of placenta and skin^d^	LMWH – prophylactic dose	4.6	Early pregnancy loss^d^	LMWH – prophylactic dose	Yes
**16**	10	2°, Thr	No	CVA, renal infarction^d^	LMWH	23.4	CVA^d^	LMWH	No (physician reported)
**18**	12	1°, Thr	Yes	Liver lesions with small-vessel thrombotic vasculopathy ^d^	LMWH	3.0	PE^d^	LMWH	No (family reported)
**19**	15	1°, Thr	Yes	Lower extremity DVT^c^	LMWH transitioned to warfarin	36.8	Portal vein thrombosis ^d^	Warfarin	No (physician reported)
						74.8	CVA^d^	Warfarin	No (physician reported)
**20**	17	1°, Thr	No	Lower extremity DVT^c^	LMWH	2.0	PE^c^	None (suspected medication interaction on LMWH)	-
						2.9	PE^c^	Warfarin (subtherapeutic) transitioning to LMWH	Yes
						4.0	PE^d^	LMWH	No (physician reported)
						11.6	PE with secondary pulmonary infarction^d^	Fondaparinux and LDA	Yes

*Pt* Patient number, *AC* Anticoagulation, *1°* Primary AOS, *2°* Secondary APS, *Obs* Obstetric, *Thr* Thrombotic, *LDA* Low dose aspirin, *TMA* Thrombotic Microangiopathy, *PE* pulmonary embolism, *DVT* Deep Vein Thrombosis, *HELLP* Hemolysis, Elevated Liver enzymes, Low Platelet count syndrome, *LMWH* Low molecular weight heparin (enoxaparin), *CVA* cerebrovascular accident. ^a^2000-2004, ^b^2005-2009, ^c^2010-2014, ^d^2015-2020

## Discussion

This case series adds to the relatively limited body of knowledge about children who develop APS and provides some important information that could inspire new ideas about how to best diagnose, manage, and research pediatric APS. For example, this series again highlights the burden of non-criteria clinical manifestations in the pediatric population and reinforces the need for pediatric-specific classification criteria. Patients who present with features such as the so-called Evans Syndrome (autoimmune hemolytic anemia and thrombocytopenia), for example, likely warrant testing for APS. Of note, the 15^th^ International Congress on Antiphospholipid Antibodies Task Force on Pediatric Antiphospholipid Syndrome published a report that highlighted the different characteristics seen in pediatric APS and also underlined the importance of large, age-stratified studies to better identify risk for thrombotic events and to test the hypothesis that adult criteria may not be as useful in children [22]. Our data also confirm that testing for ANA is not a good screening test for APS because it was only positive in 43% of all patients with APS and none of the patients with primary APS. If a diagnosis of APS is being considered, then screening should be with aPL rather than relying on ANA as a surrogate marker.

There are no pediatric-specific laboratory cut-offs for aPL with percentiles based on healthy adults. Centers may consider determining an appropriate cut-off value for positive or negative aPL results based on pediatric controls. In our series, anti-PS/PT was tested in just two patients and was positive in both. One patient presented with thrombotic microangiopathy, and their other aPL either became negative after the use of plasmapheresis and rituximab or, in the case of lupus anticoagulant, could not be relied upon because of the use of heparin products. Hence, anti-PS/PT could potentially provide useful diagnostic information in cases in which APS is strongly suspected but unable to be confirmed with current criteria lab testing. Interestingly, there have been two case reports of pediatric APS patients with positive anti-PS/PT and thrombotic microangiopathy [23, 24]. One other series of APS patients evaluating anti-PS/PT included pediatric patients and showed a significant association between test positivity and APS; it could even be used to diagnose clinical cases when other aPL were negative [25]. Further research in this area seems warranted.

Our series adds to what is known about recurrent events in pediatric APS patients. Recurrence was common in our series, involving nearly half of patients both in primary and secondary APS groups. That nearly all events occurred while a patient was not on therapeutic anticoagulation underscores the vital importance of APS patients being prescribed and staying on therapeutic levels of anticoagulation. A similar finding was seen in another pediatric case series in which there was a recurrence rate of 59%, with 80% of those events occurring when patients were not receiving therapeutic levels of anticoagulation [5]. The large pediatric APS registry had a lower recurrence rate of 19% [6], and another series of 28 children had a rate of 29% [10]. In review of the patients in our series, some physicians opted to stop anticoagulation when aPL became negative. Two such patients (patients 8 and 20, seen in Table 2 and Fig. 1) had recurrences despite aPL levels that dropped below the threshold of lab positivity over time. Although there has not been a pediatric-specific study on this topic, one study in adults found that by five years of follow-up, recurrent thrombotic events occurred in almost half of APS patients whose aPL became negative and in whom anticoagulation had been discontinued [26].

Therapeutic approaches in pediatric APS are heterogeneous and largely based on adult studies, anecdotal evidence in children, and clinicians’ experience. The largest evidence-based recommendations come from an initiative in Europe, in which treatment guidelines largely rely on descriptive studies and expert opinion; the group recommends consideration of antiplatelet agents in addition to hydroxychloroquine in patients with SLE and positive aPL; anticoagulation for venous thrombotic events when related to aPL; long-term anticoagulation in the setting of venous thrombosis and persistent aPL positivity; adequate long-term anticoagulation possibly in combination with antiaggregant therapy in the setting of arterial thrombosis and persistent aPL positivity; and consideration of elevated INR goals or alternative therapies with recurrent thrombotic events and persistent aPL positivity [27]. In our study, when evaluating the types of therapies employed, heparinoids and vitamin K antagonists (VKAs) were used most frequently, as expected. DOACs were used for some patients (4 total), most often when they became older in an effort to improve compliance. Two of the four patients had recurrent events. One recurrence occurred when the medication had recently been stopped about a week prior to the recurrence and one occurred when the patient was reported to be skipping doses (patients 8 and 13 in Table 2). One might postulate that the relatively short half-life of DOACs compared to VKAs could make patients on DOACs at particular risk for recurrence if they are not strictly adherent to their medications. Of note, there is one published case of a pediatric APS patient without recurrence on a DOAC with five months of follow-up [7], but there are few other data on this topic. Our case series found a relatively low rate of aspirin use, especially in primary APS. The primary APS group also had low utilization of hydroxychloroquine, which has been studied as a successful adjunctive therapy to anticoagulation in small studies of adult primary APS patients [28, 29] in addition to recommendations for its use in all aPL-positive patients with lupus by the 14th International Congress APS Treatment Trends Taskforce [30]. The potential use of hydroxychloroquine in pediatric APS patients underscores the potential benefits of collaboration between hematology and rheumatology, especially in refractory cases.

Our study was limited by its retrospective nature. As such, not every manifestation of interest was tested for in every patient; as an example, an echocardiogram was not performed in every patient, and as such, valve disease may have been present and missed. Some of these patients were managed by rheumatologists and others primarily by hematologists. The work-up and management were likely different due in part to the specific training and possible biases of different providers’ specialties. These patients were also collected over the course of 20 years, and the knowledge about APS has improved over that time.

The use of the DIAPS to assess for long-standing damage does show that even in pediatric APS patients, substantial morbidity can accumulate. Though some pediatric APS patients do well without acquiring new problems beyond the initial event that led to their diagnosis, others accrued significant damage over time. Although the median scores of 1.5 and 1.2 in primary and secondary pediatric APS patients, respectively, are lower than most of those previously reported in adults [26], they still indicate a growing burden of disease in a young population. Within our cohort of 21 patients, 3 accumulated severe damage based on their DIAPS score. In previous studies of adults, the DIAPS correlates inversely with quality-of-life measures [26]. Elevated scores with distinct patterns have been shown in adults with primary APS, in which damage is an early event correlated with delay in diagnosis, and in APS with SLE, in which acquired damage occurs as an accumulation over time [21]. To our knowledge, this is the first application of the DIAPS to pediatric APS patients. It may continue to be employed when evaluating pediatric APS patients to add to our understanding of the serious long-term effects of this disease in children.

## Conclusions

This case series provides additional information on certain features of pediatric APS, a relatively understudied and rare disease with significant morbidity. First, pediatric patients frequently exhibit non-criteria manifestations of APS, suggesting the need for pediatric-specific classification criteria and more widespread knowledge of these additional features when considering the diagnosis. The application of the DIAPS suggests that in some patients, there may be a significant burden of damage accrued over time. Recurrent thrombosis is common in nearly half of patients, and among those, subtherapeutic anticoagulation or anticoagulant medication non-adherence is almost universally identified. This finding emphasizes the challenges of effective anticoagulation in the pediatric population and highlights the importance of identifying other effective treatments in APS. Recurrent events occurred even when some patients’ aPL titers fell below positive thresholds, so this may not be a reliable indication as to when it might be safe to stop anticoagulation. With regards to lab testing for APS, anti-PS/PT could be considered as an additional aPL lab test in patients for whom clinical suspicion of APS is high. ANA may not be positive in APS, particularly primary APS, and so it should not be used as a screening test for APS in pediatric patients. The clinical characteristics, laboratory features, and outcomes described in this case series add to what is known about pediatric APS and may suggest areas requiring further study.

## Data Availability

The datasets generated and analyzed during the current study are not publicly available because the sharing could compromise individual privacy, but deidentified datasets are available from the corresponding author on reasonable request.

## Abbreviations

ANA: Antinuclear antibody
aPL: Antiphospholipid antibody
APS: Antiphospholipid syndrome
CAPS: Catastrophic APS
DIAPS: Damage index in patients with thrombotic antiphospholipid syndrome
DOAC: Direct oral anticoagulant
DRVVT: Dilute Russell’s viper venom test
GPL/MPL: IgG phospholipid units/IgM phospholipid units. One unit is 1 µg of antibody.
IVIG: Intravenous immunoglobulin
PS/PT: Phosphatidylserine/prothrombin
SDI: SLICC/ACR Damage Index
SLE: Systemic lupus erythematosus
VKA: Vitamin K antagonist
